# Comparison of the effectiveness of autologous grafts for anterior cruciate ligament reconstruction: A protocol for an overview of systematic review and network meta-analysis

**DOI:** 10.1097/MD.0000000000022832

**Published:** 2020-10-23

**Authors:** Jia-Xin Jin, Peng-Zhong Fang, Zhi-Wei Hu, Jin-Lei Chen, Rui-Rui Wang, Xin Wang

**Affiliations:** a Department of Orthopedics, The Second Hospital of Lanzhou University; b The First Clinical Medical College of Lanzhou University, Lanzhou, Gansu Province; c Department of Orthopedics, Changzheng Hospital, Naval Medical University, Shanghai, China.

**Keywords:** anterior cruciate ligament reconstruction, autograft, overview, systematic review, network meta-analysis

## Abstract

**Background:**

Anterior cruciate ligament rupture is a common motor system injury, and the most effective treatment is anterior cruciate ligament reconstruction (ACLR). Choosing the right graft is an important factor to ensure the success of the surgery. Current research shows that the clinical effect of autologous ligaments is better than that of allogeneic ligaments and artificial ligaments. However, there are differences between the autogenous ligaments, and how to choose them is still controversial. This study evaluated the published systematic reviews on the efficacy of different autologous ligament grafts in ACLR, and based on this, conducted a network meta-analysis of related randomized controlled trials.

**Methods:**

We searched 8 international and Chinese databases including PubMed, Embase, Web of Science, and Cochrane Library. The methodological quality of systematic reviews will be evaluated by Assessing the Methodological Quality of Systematic Reviews-2 (AMSTAR2) measurement tool. Cochrane's risk of bias tool will be used to assess the risk of bias of included randomized controlled trials, and the Grading of Recommendations Assessment, Development, and Evaluation (GRADE) approach will be used to evaluate the evidence quality. Network meta-analysis will be applied to evaluate the therapeutic effect of different autologous grafts. The main outcome measures are IKDC score, clinical failure rate, Lachman test, Lysholm score, and the incidence of complications. Odds ratio and its 95% confidence interval will be used to synthesize the dichotomy results, while the mean difference and 95% confidence interval of continuous variables will be used for continuous variables.

**Results:**

This study will provide comprehensive evidence for the application of autologous grafts in ACLR.

**Conclusion:**

The results of this study will help clinicians make appropriate decisions.

**Protocol Registration number:**

INPLASY202090061.

## 1 Introduction

Anterior cruciate ligament is an intra-articular ligament that originates from the posterolateral side of the intercondylar notch and extends forward to insert into the intercondylar eminence.^[1]^ It is one of the most important ligaments to maintain the normal function and stability of the knee joint. Its main function is to limit the tibial forward movement and knee varus and valgus in the state of extension.^[1]^ Anterior cruciate ligament (ACL) rupture is mainly noncontact injuries, which mainly affect women, young people, and athletes.^[2]^ ACL injuries account for more than 50% of all knee injuries and affecting exceed 200,000 people in the United States each year and the prevalence is on the rise, with direct and indirect costs exceeding 7 billion US dollars each year.^[2–4]^ According to the patient's injury situation and personal wishes, rehabilitation or surgical treatment can be selected. With the development of surgical technology and research, anterior cruciate ligament reconstruction (ACLR) has gradually become the mainstream treatment of ACL rupture. From the long-term perspective, compared with rehabilitation therapy, ACLR can save more than 50,000 dollars per patient on average.^[5]^

Anterior cruciate ligament injury can lead to decreased knee stability, and increase the risk of meniscus injury and early osteoarthritis.^[6]^ The purpose of ACLR is to restore the normal anatomical structure of the knee joint and reconstruct biological and mechanical stability.^[6]^ The risk of secondary meniscus tear was significantly lower in patients treated with ACLR than in patients with nonsurgical treatment after ACL tear.^[7]^ There are many kinds of grafts for ACLR, including autogenous ligament, allogeneic ligament, and artificial ligament. Among them, autogenous grafts are the most widely used. The commonly used autogenous grafts include bone-patellar tendon-bone, quadriceps femoris, hamstring tendon, peroneus longus tendon, etc.^[8]^

The choice of graft is a critical factor affecting the surgical effect of ACLR because the biological and mechanical properties of different types of grafts are different.^[9]^ Overall, the effect of autologous tendon transplantation is better than that of allograft and artificial ligament, and there are differences among each autograft. For many years, bone-tendon-bone autograft has been regarded as the gold standard for ACLR.^[10]^ However, in recent years, hamstring tendon, quadriceps femoris tendon, and peroneus longus tendon autograft have also been used for ACLR and have shown well clinical results.^[11,12]^ Although many kinds of autografts have shown good applicability, how to select it is still controversial.^[13]^

Systematic review and meta-analysis provide important information for clinical decision-making and are also the main sources of evidence in the development of clinical guidelines.^[14,15]^ The standard meta-analysis can only provide a comparison between the 2 interventions, which has certain limitations and cannot fully answer a clinical question.^[16]^ However, the network meta-analysis (NMA) can evaluate the relative effectiveness of various interventions and synthesize evidence in the whole randomized trial network.^[16]^ Although a certain number of systematic reviews (SRs) have been published on autologous tendon transplantation in ACLR, there is still a lack of methodological quality evaluation. An NMA can also help to comprehensively analyze the differences between autografts and provide references for clinical decision-making and future research.

## 2 Method

### 2.1 Design and registration

This study will conduct an overview of SRs of the efficiency of the autologous ligament grafts in ACLR and a network meta-analysis will be performed on the included RCTs. Because this is a literature-based study, ethical approval is not required. This study will follow the Preferred reporting items for systematic review and meta-analysis protocols (PRISMA-P) statement for reporting our overview.^[17]^ This protocol has been registered on the International Platform of Registered Systematic Review and Meta-analysis Protocols (INPLASY) database (protocol number: INPLASY202090061, Doi: 10.37766/inplasy2020.9.0061).

### 2.2 Data sources and search strategy

We comprehensively searched the databases including PubMed, Embase, Web of Science, Cochrane Library, Chinese biomedical literature database (CBM), Chinese National Knowledge Infrastructure (CNKI), and Wanfang Database. There are no restrictions on the search. All searches were until August 31, 2020. Grey literature and references included in the literature will also be reviewed. We combined medical subject headings (MeSH) and free words with boolean logical operators to construct a search strategy. The search strategies were formulated separately according to the characteristics of each database. Tables 1 and 2 show the search process of PubMed and Web of Science, respectively.

**Table 1:**
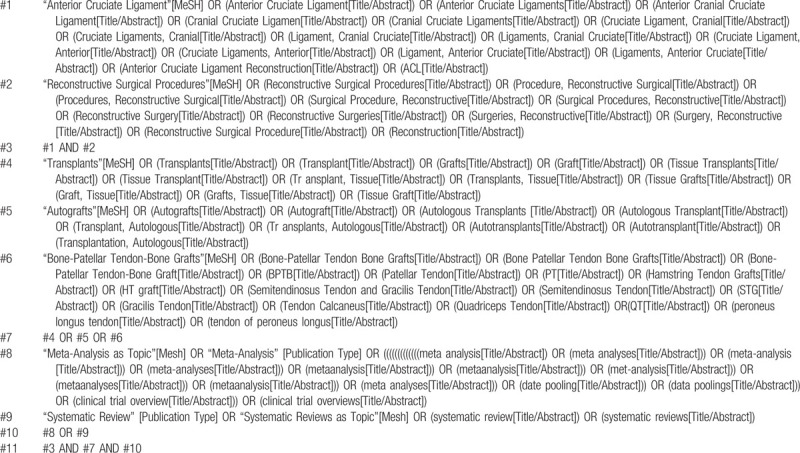
Searching strategy in PubMed.

**Table 2:**
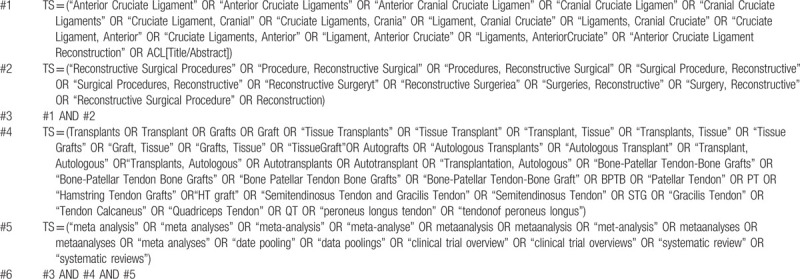
Searching strategy in Web of Science.

### 2.3 Study selection

#### 2.3.1 Type of study

Systematic reviews and meta-analyses that meet the inclusion and exclusion criteria and the RCTs included in them.

#### 2.3.2 Inclusion criteria

(1) Participations: Clinical diagnosis of anterior cruciate ligament rupture, the first time to receive anterior cruciate ligament reconstruction, and the patient's age, gender, nationality, race, injury time is not limited
(2) Intervention: All types of autologous tendon grafts, including bone-patellar tendon-bone, quadriceps tendon, hamstring tendon, peroneus longus tendon, etc
(3) Comparator: Different types of autologous ligament grafts
(4) Outcomes: The main outcome indicators include IKCD score, clinical failure rate (including revision surgery, graft rupture, +2 pivot shift or higher, and side-to-side arthrometer difference >5 mm), Lachman test, Lysholm score, instrument laxity test, joint range of motion, Tegner score, complications.^[18]^
(5) Peer-reviewed articles published in Chinese or English
(6) SRs including RCTs, meta-analysis results, and consistent with established PICO.

#### 2.3.3 Exclusion criteria

(1) Animal research
(2) Letters, conference papers
(3) Descriptive research
(4) Full text is not available
(5) Repeated publications
(6) Important data are missing and cannot be obtained after contacting the authors.

### 2.4 Data collection

#### 2.4.1 Literature screening

**Figure 1:**
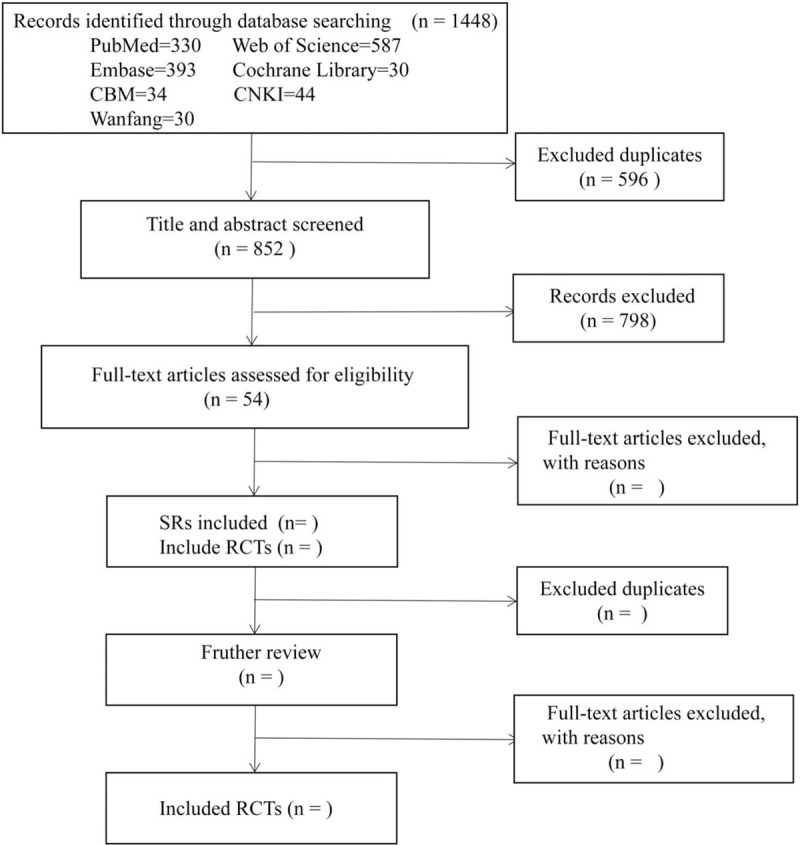
A flow diagram of the literature search and selection process. CBM = Chinese Biomedical Literature Database, CNKI = Chinese National Knowledge Infrastructure.

All the searched literature were imported into Endnote9.0 software. We identified 1448 records through database searching, removed 596 duplicate records, and then excluded 798 records by reading the title and abstract. There are 54 items to be further screened by reading the full text. All the screening process was completed by 2 reviewers independently. The difference will be determined after discussion with the third reviewer. The RCTs included in the SRs will be extracted, and the eligible RCTs will be used to conduct a network meta-analysis after eliminating the repetitive literature. The process of literature screening will be shown by a flow chart (Fig. 1).

#### 2.4.2 Data extraction

The data extraction table was designed in advance, and the extracted information for SRs included:

(1) General characteristics of literature: a. Title b. First author c. Year of publication d. Formulation country e. Publication Journal f. Source of funds
(2) Methodological characteristics: a. Whether to conduct a comprehensive search; b. The number and name of the retrieved databases; c. Inclusion and exclusion criteria; d. Inclusion of literature quality evaluation methods
(3) Included research: a. The number of included studies b. The total number of patients c. Gender, age, and ethnic characteristics d. Intervention and comparator e. Outcome indicators f. Main conclusions
(4) Others

For the selected RCTs, the extracted information included:

(1) General characteristics: a. Title b. First author c. Year of publication d. Formulation country e. Publication Journal f. Source of funds
(2) Methodological characteristics: a. The number of patients b. Source of patients c. Characteristics of patients d. Details of intervention and control measures e. Outcome indicators f. Main conclusions
(3) Others

Five qualified documents will be used for data preextraction and the extraction table will be revised and improved. All the data extraction process was completed by 2 reviewers independently. If there were divergence of views, they would be discussed and solved with the third reviewer.

### 2.5 Methodological quality assessment of included SRs

The quality of published SRs may be quite different, and it is necessary to evaluate it. Assessing the Methodological Quality of Systematic Reviews-2 (AMSTAR2) is an instrument for rigorously evaluating the systematic review of randomized controlled clinical trials which contains 16 items and 7 of them are critical items.^[19]^ It can be evaluated as “Yes,” “Partial Yes,” “No” or “No meta-analysis conducted.” Furthermore, based on critical items, the overall confidence in the results of SRs can be divided into 4 levels: high, moderate, low, and critically low.^[20]^ The evaluation process is completed independently by 2 reviews, and if there is a disagreement, it will be discussed and resolved with the third.

### 2.6 Evidence quality of outcome measures

We will use the Grading of Recommendations Assessment, Development, and Evaluation (GRADE) to assess the quality of evidence. The limitations of research, consistency of effect, imprecision, indirectness, and publication bias will be mainly considered.^[15,21]^

Before the assessment, the evidence quality of all results is assumed to be “high” and will eventually be evaluated as “high,” “moderate,” “low” or “very low.”^[21]^

### 2.7 Assessment of risk of bias of included RCTs in identified SRs

The Cochrane's risk of bias tool will be used to assess the bias risk of included RCTs. Bias risk assessment includes 6 aspects, including selection bias, performance bias, detection bias, incomplete outcome data, reporting bias, and other biases.^[22]^ For each result, it will be assessed according to the evaluation criteria as low risk of bias, high risk of bias, and uncertain bias or lack of relevant information. If important information is missing or incomplete, we will try to contact the author to obtain it.

### 2.8 Data synthesis

#### 2.8.1 Basic characteristics

We will conduct a descriptive analysis of the included SRs and present them in a table.

#### 2.8.2 Evidence map

We will present the differences in the methodological quality of the SRs by drawing bubble charts, which also includes information on the number of RCTs contained in the SRs and the types of interventions.

#### 2.8.3 Network meta-analysis of included RCTs

The network evidence map will be drawn to compare the relationship between different interventions directly or indirectly. The odds ratio and its 95% confidence interval will be used to synthesize the results of the dichotomy, while the mean difference and its 95% confidence interval will be used for continuous variables. *P* < .05 was considered to be statistically significant. Heterogeneity analysis will be conducted for the studies included, and the *I*^2^ value represents the strength of heterogeneity. If *I*^2^ ≤ 50%, it means that there is a low heterogeneity and the fixed effects model will be adopted. If *I*^2^ ≥ 50%, it means that the heterogeneity is high, and the source of the heterogeneity will be further analyzed, and the random-effects model will be adopted after the heterogeneity is excluded. Studies with high heterogeneity will be subjected to subgroup analysis or sensitivity analysis. The Egger test and funnel chart will be used to assess potential publication bias.^[23,24]^ NMA combines direct and indirect evidence within the Bayesian framework and uses WinBUGS statistical software (version 1.4.3) to implement Markov Chain Monte Carlo (MCMC) method. The SUCRA graph predicts the efficacy of each graft. SUCRA is a ratio, expressed as the percentage of the efficiency of intervention to the result.^[25]^ When the treatment effect is better, the value is closer to 100%, and vice versa, the value is closer to 0%.^[25]^ The node-splitting model assesses the inconsistency of this network meta-analysis. A significance level of less than 0.05 is interpreted as inconsistent evidence.^[26]^

### 2.9 Sensitivity analysis and subgroup analysis

According to the results of data extraction and analysis, we will analyze different subgroups such as gender, age, and different surgical methods, etc. If possible, we will do some additional subgroup analyses based on the results of heterogeneity and inconsistency. If the evidence is sufficient, we will conduct a sensitivity analysis to exclude those important data missing, low quality or small studies, and high risk of bias trials to ensure the stability of the results.

## 3 Discussion

The ACL rupture is attracting increasing attention due to its high incidence rate and serious health impact. Arthroscopic surgery has the advantages of low trauma and fewer complications and has gradually become a common treatment for ACL rupture.^[27]^ Among all the factors affecting the clinical effect of ACLR, the choice of graft is undoubtedly the most critical and controversial topic. Of course, how to choose a graft is not a single problem, but should consider a variety of factors, including the patient's age, gender, daily activity, functional needs, clinical failure rate, and complications, and make an individual selection after comprehensive evaluation of patients. This study will make a comprehensive comparison of the effects of different autografts in ACLR. The overview of SRs will help to clarify their methodological quality, and the research results will provide a reference for clinical selection. Although there are many kinds of autografts available, further research is needed to optimize the treatment and obtain better clinical effects.

## Acknowledgment

We thank Dr. Jinhui Tian, Dr. Meili Yan, and Dr. Yamin Chen for his assistance on the methodology of this study.

## Author contributions

**Conceptualization:** Jia-Xin Jin, Xin Wang.

**Data curation:** Peng-Zhong Fang, Zhi-Wei Hu, Jin-Lei Chen, Rui-Rui Wang.

**Methodology:** Xin Wang.

**Software:** Jin-Lei Chen, Rui-Rui Wang.

**Writing – original draft:** Peng-Zhong Fang, Zhi-Wei Hu.

**Writing – review & editing:** Jia-Xin Jin.
